# First isolation of dengue virus from Lao PDR in a Chinese traveler

**DOI:** 10.1186/1743-422X-10-70

**Published:** 2013-03-04

**Authors:** Xiaofang Guo, Qiumin Zhao, Chao Wu, Shuqing Zuo, Xiaoai Zhang, Na Jia, Jiangyun Liu, Hongning Zhou, Jiusong Zhang

**Affiliations:** 1 State Key Laboratory of Pathogen and Biosecurity, Beijing Institute of Microbiology and Epidemiology, Beijing, People’s Republic of China; 2 Yunnan Institute of Parasitic Diseases, Pu’er, Yunnan, People’s Republic of China; 3 Mengla County Center for Diseases Control and Prevention, Xishuangbanna, People’s Republic of China

**Keywords:** Dengue virus, Isolation, Envelope protein, Phylogenetic analysis

## Abstract

**Background:**

Epidemic dengue activity has been demonstrated in several southern regions of China, but not in Yunnan province, which borders countries in Southeast Asia where dengue is endemic. Many dengue cases imported from Southeast Asia to Yunnan have been reported, but dengue virus (DENV) has not been isolated from any patients. This study is the first to report the isolation of DENV from a Chinese traveler returning to Yunnan from Lao PDR.

**Findings:**

A serum sample was collected from a patient presenting with a febrile illness who returned from Lao PDR in 2009 and was used to inoculate *Aedes albopictus* C6/36 cells for viral isolation. The viral isolate was identified using reverse transcription-polymerase chain reaction, and phylogenetic analyses based on the full E sequence were performed using Clustalx 1.8 software. The analyses detected DENV genome, and thus, a DENV isolate was obtained from the patient’s serum sample. The new DENV isolate was grouped into genotype Asia 1, serotype 2. The viral E protein shared the greatest nucleotide sequence identity (99.6%) with the D2/Thailand/0606aTw strain isolated from Thailand in 2006 and demonstrated 94.3% to 100% identity with the predicted amino acid sequence of other DENV 2 strains.

**Conclusions:**

Our findings indicate that DENV serotype 2 is circulating in Lao PDR, and surveillance of patients suspected of infection with dengue should be conducted not only by a serological test but also by pathogenic detection methods.

## Introduction

Dengue virus (DENV), a member of the *Flavivirus* genus of the *Flaviviridae* family, has four distinct serotypes (DENV 1 to 4) that cause a spectrum of disease ranging from asymptomatic, mild, undifferentiated fever and classical dengue fever to the severe disease known as dengue hemorrhagic fever/dengue shock syndrome [1]. Dengue has emerged as a major mosquito-borne viral disease in tropical and subtropical regions over the past 20 years. The disease is prevalent in Africa, the Americas, the Eastern Mediterranean, Southeast Asia and the Western Pacific. An estimated 2.5 billion people live in areas where dengue is endemic, and the annual incidence of the disease in these areas is approximately 50 million cases. In the last 50 years, the incidence of the disease has increased >30-fold due to increasing geographic expansion and, in the present decade, a move from urban to rural areas [2,3].

In China, epidemic dengue activity has been detected in the southern regions, primarily the provinces of Guangxi, Guangdong, Fujian, Zhejiang and Hainan [4]. From 1978 to 2008, a number of dengue outbreaks were reported, comprising a total of 655,324 cases and resulting in 610 deaths [5]. Yunnan Province, located in southern China, has a tropical to subtropical climate and borders Vietnam, Lao PDR and Myanmar, countries in which dengue is endemic [6-8]. Although no dengue outbreak has been recorded, imported cases have frequently been reported in Yunnan. In 2008, 77 imported cases and 12 indigenous cases (on the border with Myanmar) were reported, all of which were diagnosed by a serological test [9].

In the present study, we report the isolation of a DENV 2 strain from a patient with a febrile illness who returned from Lao PDR in 2009. This is the first report of DENV isolated from an individual who had traveled to Lao PDR.

## Materials and methods

This study was performed after consultation with the patient and receipt of written consent. All study-related information was acquired and used anonymously. The Institutional Review Board of the Beijing Institute of Microbiology and Epidemiology approved the research involving human subjects.

**Figure 1:**
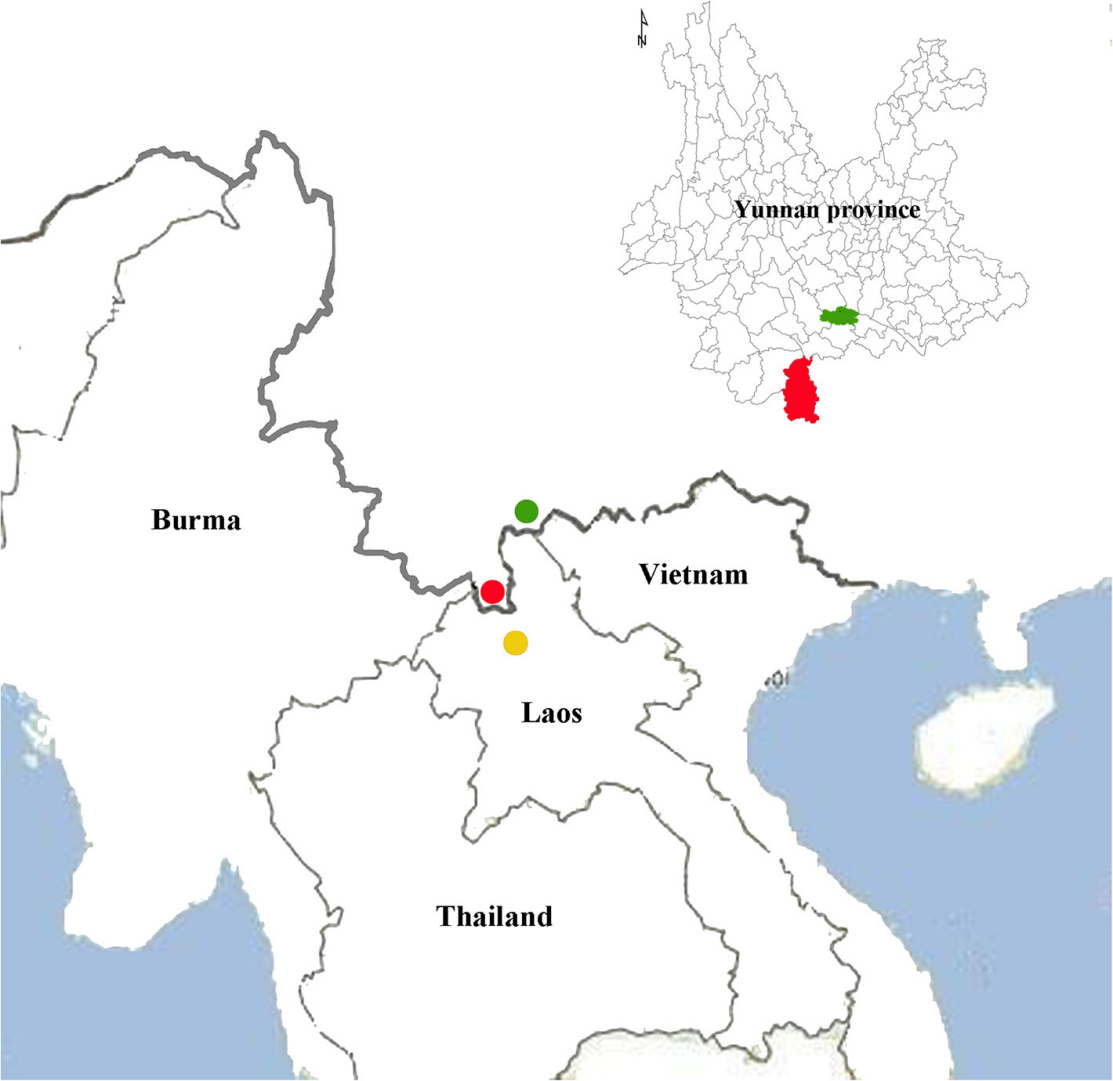
**Map of the Yunnan province and surrounding regions.** Yellow dot indicates location of DENV-infected site. Red dot and shading indicate location of diagnosed site. Green dot and shading indicate location of living site of the patient.

A 22-year-old man with symptoms of persistent fever (>39°C) and headache visited a local health center in China at the China-Lao PDR border on October 16^th^, 2009. The patient had been living in Muong Xay Town, Lao PDR, which is located in the north of Lao PDR, for six months as a part-time worker before his visit to the health center (Figure 1). Muong Xay Town and the China-Lao PDR border are approximately 100 kilometers apart. The onset of illness occurred on October 15^th^, 2009. Because there was no effective treatment available in Muong Say Town, the patient returned to Yunnan to visit a Chinese doctor one day after his symptoms began. In accordance with a routine surveillance program, a serum sample was collected from the patient on the day he visited the doctor, and it was tested for immunoglobulin (Ig) G and IgM antibodies to DENV using a rapid test card (OneStep Dengue RapiCard™ InstaTest, Cortez Diagnostics Inc., Calabasas, California, USA)*.* This test is the standard method used for the rapid diagnosis of DENV infections. The test was negative. No skin rashes or other systemic symptoms were apparent. The patient was hospitalized as a suspected case of dengue fever for three days until defervescence.

DENV genome in serum was detected by reverse transcription-polymerase chain reaction (RT-PCR). Briefly, total RNA was extracted from the patient’s serum sample using Trizol and was reverse-transcribed using random hexamers to obtain the first-strand cDNA. The PCR was performed with a pair of DENV universal primers, which amplified the partial capsid-premembrane genes (511 bp). A semi-nested PCR was then performed using the type-specific primers described by Lanciotti [10]. The PCR products were directly sequenced using an automated DNA sequencer (ABI PRISM 373, Perkin-Elmer, USA). The DNA was sequenced using both forward and reverse primers to verify the sequences. The sequences were then subjected to a BLAST search.

The serum specimen that was positive for DENV was diluted 1:10 in RPMI-1640 medium containing 2% fetal calf serum and cultured with *Aedes albopictus* C6/36 cells at 32°C for viral isolation. The cultures were examined daily for evidence of a virus-induced cytopathic effect (CPE). The culture supernatant was filtered with a 0.22-μm filter and then blind-passaged. The isolate was then identified using a DENV-specific RT-PCR as described above.

A pair of primers (Den2-EF: AAC ATG GAT GTC ATC AGA AGG; Den2-ER: CCA ATC TTG TTA CTG AGC GG) was used to amplify the envelope (E) gene of the virus from a cell culture infected with the isolate. The E sequence generated in this study was aligned with 32 reference sequences downloaded from GenBank, including all four serotypes of DENV. A pairwise analysis between the strains was performed using the DNAstar program. Multiple sequence alignments were carried out using Clustalx 1.8 software [11]. Phylogenetic trees were generated using the Bayesian Metropolis-Hastings Markov Chain Monte Carlo (MCMC) treesampling methods implemented by Mr. Bayes 3.1 software. We used the GTR model as the evolutionary model with gamma distributed rate variation across sites and a proportion of invariable sites [12]. The run was stopped when the standard deviation of split frequencies was below 0.01. A DENV 3 strain H87 sequence was used as an out-group control.

## Results and discussion

**Figure 2:**
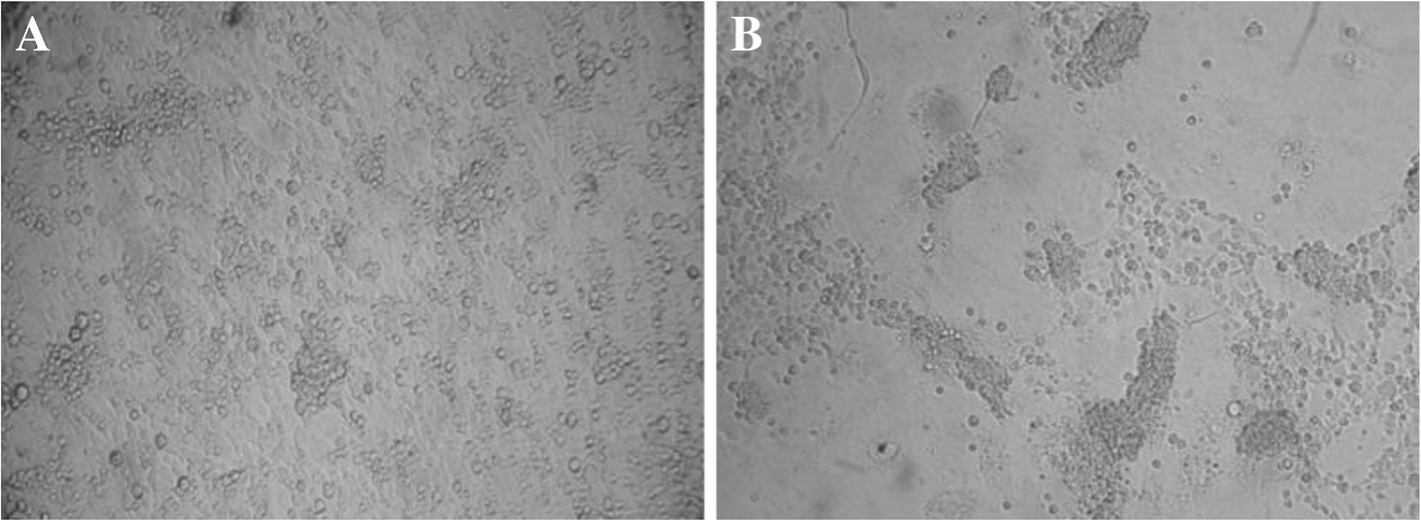
**Viral isolate MLDENV-09-induced cytopathic effect (CPE) on C6/36 cells. A**: normal C6/36 cells; **B**: virus-infected C6/36 cells.

The patient’s serum was positive for DENV genome as determined by RT-PCR using DENV universal primers. After amplification with type-specific primers, a 119-bp nucleotide segment was obtained, which matched serotype 2 of DENV. When the serum specimen was used to inoculate C6/36 cells, a significant CPE was observed in the second passage on day 6 after inoculation. Cell fusion characterized the CPE (Figure 2). The viral isolate, designated as MLDENV-09, was positive for DENV RNA by RT-PCR with the universal primers. The isolate was identified as DENV serotype 2 by semi-nested PCR using the type-specific primers and a BLAST search. Furthermore, the full E sequence of 1 485 bp was obtained and deposited into the GenBank database under the accession number JQ815199.

**Figure 3:**
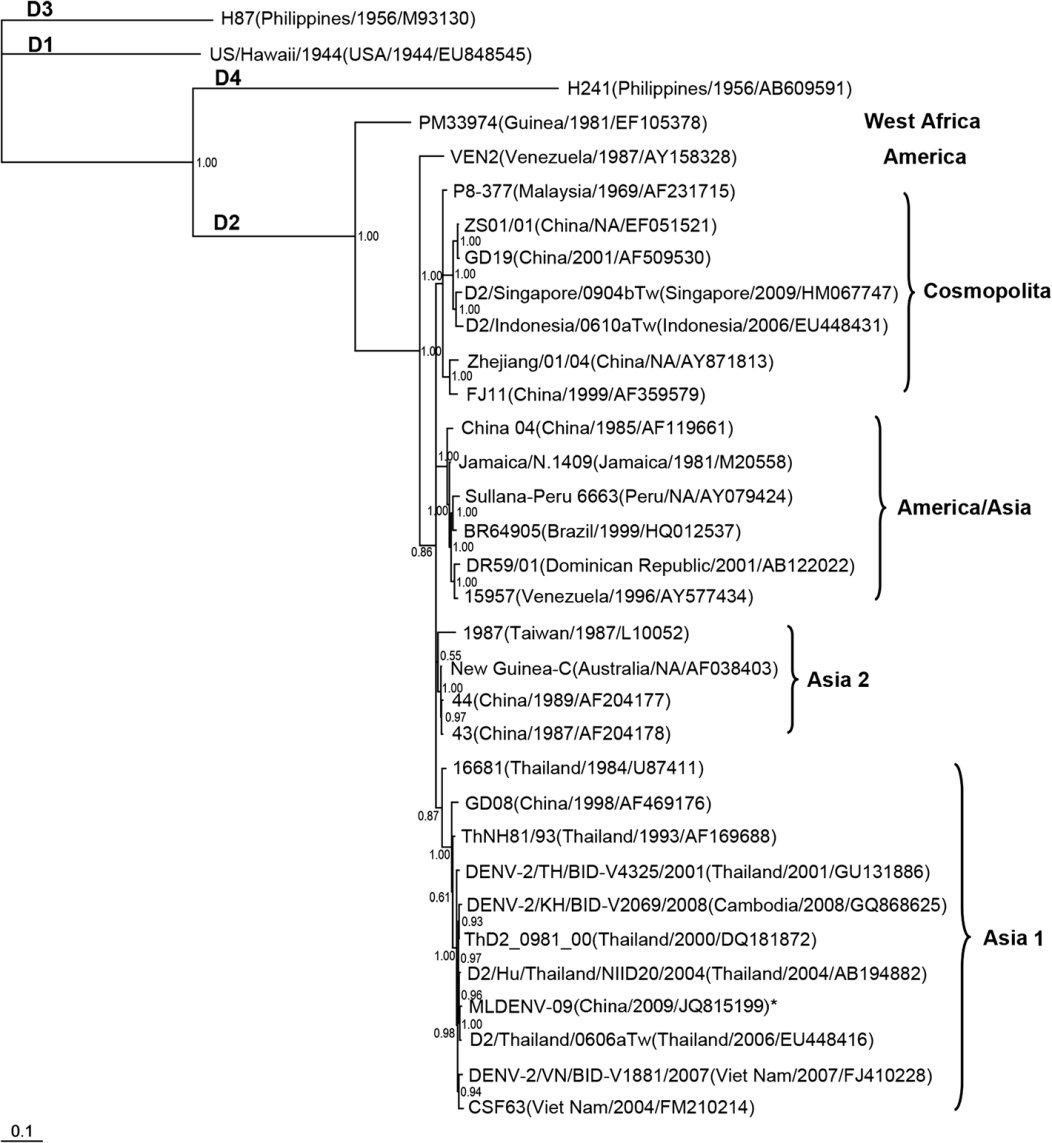
**Phylogenetic tree based on the full E gene of the reference DENV strains.** DENV 3 was used as out-groups. The strains are denoted by the strain name, country of isolation, year of isolation and GenBank accession number. “NA” means “not available.” DENV serotypes 1–4 are shown as D1–D4, respectively. An asterisk is used to denote the new isolate identified in this study.

The phylogenetic tree based on the full E sequences of 30 DENV 2 and the reference DENV 1, 3 and 4 strains is shown in the Figure 3. Separately from the US/Hawaii/1944, H87 and H241 strains, which are recognized as DENV serotypes 1, 3 and 4, respectively, the 30 sequences of DENV 2 were grouped into three distinct phylogenetic groups with high bootstrap support. The PM33974 and VEN2 strains, which belonged to West Africa and America genotypes, respectively, were classified into their own clade. Another cluster consisted of 28 closely related sequences that branched into four sub-clades generally recognized as the Asia 1, Asia 2, America/Asia and Cosmopolitan genotypes. The newly isolated MLDENV-09 strain was grouped into the Asia 1 clade with 10 other strains isolated from 1984 to 2008, including 9 from Southeast Asia (Thailand, Vietnam and Cambodia) and one Chinese sequence, GD08/98.

When compared with the other Asia 1 strains used in this study, the new isolate, MLDENV-09, had 95.6%-99.6% nucleotide sequence identity and 97%-100% amino acid sequence identity. The MLDENV-09 strain had the greatest nucleotide sequence similarity (99.6%) with the D2/Thailand/0606aTw strain isolated from Thailand in 2006, and it had 100% amino acid sequence identity with two Thai strains, ThD2_0981_00 and D2/Hu/Thailand/NIID20/2004.

Many cases of dengue with pathogenic presentation have been imported from Southeast Asia to China, but the introduction of dengue from Lao PDR has never been reported prior to the case described here [13,14]. To our knowledge, this is the first isolation of DENV from Lao PDR. The phylogenetic analysis based on the viral E gene, which is generally used to identify the DENV genotype [15], showed that the new isolate belonged to serotype 2 of DENV and was classified into the Asia 1 genotype (Figure 3).

No dengue endemic has been detected in Yunnan to date. However, there is a greater potential for a dengue epidemic in Yunnan under the current circumstances, as people and materials frequently transfer during frontier trade and tourism on the border of Yunnan. Moreover, one of the competent vectors for DENV, the *Aedes albopictus* mosquito, is distributed throughout the Yunnan province [16]. Therefore, if DENV invades, an outbreak could occur in Yunnan. Through surveillance mechanisms, we detected an imported dengue case in a person returning from Lao PDR who was experiencing the acute-phase of infection with DENV, when specific antibodies were not yet apparent, but a viral isolate was obtained.

In conclusion, our findings indicate that laboratory-based surveillance for dengue is of great importance to prevent disease outbreaks. Sentinel clinics located in high-risk areas should reinforce surveillance of febrile cases using serological detection, viral nucleotide screening and virus isolation.

## Abbreviations

DENV: Dengue virus; RT-PCR: Reverse Transcription-Polymerase chain reaction; CPE: Cytopathic Effect; E: Envelope

## Competing interests

The authors declare that they have no competing interests.

## Authors’ contributions

JZ and HZ conceived and designed the experiments. XG and QZ performed the experiments. SZ, XZ and NJ analyzed the data. CW performed the serological test. JL collected the information and serum sample of the patient. XG and JZ wrote the paper. HZ was responsible to contact with the local health center where the patient visited. All authors read and approved the final manuscript.
